# Impact of Oxidant Gases on the Relationship between Outdoor Fine Particulate Air Pollution and Nonaccidental, Cardiovascular, and Respiratory Mortality

**DOI:** 10.1038/s41598-017-16770-y

**Published:** 2017-11-27

**Authors:** Scott Weichenthal, Lauren L. Pinault, Richard T. Burnett

**Affiliations:** 1 Department of Epidemiology, Biostatistics, and Occupational Health, McGill University, Montreal, QC Canada; 2 Air Health Effects Science Division, Health Canada, Ottawa, ON Canada; 3 Health Analysis Division, Statistics Canada, Ottawa, ON Canada; 4 Population Studies Division, Health Canada, Ottawa, ON Canada

## Abstract

Outdoor fine particulate air pollution (PM_2.5_) is known to increase mortality risk and is recognized as an important contributor to global disease burden. However, less is known about how oxidant gases may modify the chronic health effects of PM_2.5_. In this study, we examined how the oxidant capacity of O_3_ and NO_2_ (using a redox-weighted average, O_x_) may modify the relationship between PM_2.5_ and mortality in the 2001 Canadian Census Health and Environment Cohort. In total, 2,448,500 people were followed over a 10.6-year period. Each 3.86 µg/m^3^ increase in PM_2.5_ was associated with nonaccidental (Hazard Ratio (HR) = 1.095, 95% CI: 1.077, 1.112), cardiovascular (HR = 1.088, 95% CI: 1.059, 1.118), and respiratory mortality (HR = 1.110, 95% CI: 1.051, 1.171) in the highest tertile of O_x_ whereas weaker/null associations were observed in the middle and lower tertiles. Analysis of joint non-linear concentration-response relationships for PM_2.5_ and O_x_ suggested threshold concentrations between approximately 23 and 25 ppb with O_x_ concentrations above these values strengthening PM_2.5_-mortality associations. Overall, our findings suggest that oxidant gases enhance the chronic health risks of PM_2.5_. In some areas, reductions in O_x_ concentrations may have the added benefit of reducing the public health impacts of PM_2.5_ even if mass concentrations remain unchanged.

## Introduction

Numerous studies have documented the relationship between long-term exposure to outdoor fine particulate air pollution (PM_2.5_) and mortality and these pollutants are recognized as important contributors to global disease burden^1^. However, PM_2.5_ is only one component of complex air pollution mixtures and it is not clear how/if the magnitude of PM_2.5_ health risks depend on concentrations of oxidant gases. This is an important question as populations are simultaneously exposed to *both* PM_2.5_ and oxidant gases (e.g. O_3_ and NO_2_) but it is not clear how the chronic health risks PM_2.5_ may depend on these other pollutants.

Crouse *et al*.^2^ recently examined multi-pollutant and cumulative-risk models for the relationship between PM_2.5,_ NO_2_, O_3_, and non-accidental mortality and noted positive associations for all three pollutants; however, this study did not specifically evaluate how these oxidant gases may modify the chronic health effects of PM_2.5_. Nevertheless, existing evidence suggests that such effect modification is biologically plausible. For example, elevated O_3_ concentrations are known to deplete anti-oxidants in the lung lining fluid^3^ and increase the permeability of the lung epithelium^4–7^. Therefore, PM_2.5_ exposures may be more harmful in regions with increased levels of oxidant gases owing to decreased oxidant defense at the initial site of pulmonary deposition as well as a more permeable lung epithelial barrier.

In this study, we evaluated the extent to which oxidant gases may modify associations between outdoor PM_2.5_ mass concentrations and non-accidental, cardiovascular, and respiratory mortality in a large population-based cohort of Canadians. Our primary interest was in evaluating how the *combined* oxidant capacity of these gases (calculated using their redox-weighted average (O_x_), described below) may potentiate PM_2.5_ health effects.

## Results

Participant characteristics are summarized in Table 1. In total, 233,340 non-accidental, 77,000 cardiovascular, and 21,100 respiratory deaths were observed during the 10.6-year follow-up period. As expected, residential estimates of outdoor air pollution concentrations across Canada were low (Table 2) with PM_2.5_ concentrations ranging from approximately 1 to 20 µg/m^3^ with a mean value of 7.37 µg/m^3^. PM_2.5_ and O_x_ were moderately correlated (r = 0.66). Spatial variations in NO_2_, O_3_, O_x_, and PM_2.5_ across Canada are shown in Supplemental Figures S1–S4.

**Table 1:** Descriptive statistics of the 2001 CanCHEC analytical sample, with Cox proportional hazard ratios among levels of each covariate.

Covariate	Persons	HR^†^	95% CI
All	2,448,500	—	—
**Sex**			
Male	1,185,500	—	—
Female	1,263,000	—	—
**Age group (years)**			
25 to 29	222,100	—	—
30 to 39	574,400	—	—
40 to 49	634,900	—	—
50 to 59	446,000	—	—
60 to 69	286,700	—	—
70 to 79	206,200	—	—
80 to 89	78,100	—	—
**Visible minority status**			
White or Aboriginal^§^	2,419,700	1.000	—
Visible minority	28,800	0.868	0.825–0.913
**Marital status**			
Single^§^	323,000	1.000	—
Common-law	294,700	0.788	0.769–0.807
Married	1,491,200	0.676	0.666–0.686
Separated	59,700	0.996	0.966–1.026
Divorced	140,700	1.006	0.985–1.028
Widowed	139,200	0.898	0.884–0.913
**Educational attainment**			
Not completed high school^§^	704,400	1.000	—
High school with/without trades certificate	887,600	0.803	0.795–0.810
Post-secondary non-university	473,600	0.670	0.660–0.680
University degree	382,900	0.551	0.542–0.561
**Income adequacy quintile**			
1st quintile - lowest^§^	373,600	1.000	—
2nd quintile	465,100	0.816	0.807–0.825
3rd quintile	509,900	0.711	0.702–0.720
4th quintile	537,400	0.633	0.625–0.642
5th quintile - highest	562,600	0.536	0.528–0.543
**Labour force status**			
Employed^§^	1,580,900	1.000	—
Unemployed	103,800	1.608	1.559–1.659
Not in labour force	763,800	1.944	1.917–1.971
**Population Centre Size** ^‡^			
Rural area^§^	641,800	1.000	—
Small population centre (1,000 to 29,999)	387,000	0.982	0.968–0.996
Medium population centre (30,000 to 99,999)	230,700	0.980	0.965–0.995
Large population centre (100,000 or more)	1,151,400	0.982	0.972–0.993
not assigned (dummy variable)	37,600	—	—
**Airshed** ^‡^			
Western^§^	265,800	1.000	—
Prairie	288,600	1.083	1.062–1.104
West Central	164,900	1.115	1.091–1.139
East Central	1,376,300	1.035	1.021–1.049
South Atlantic	268,200	1.060	1.041–1.079
Northern	42,500	1.121	1.067–1.178
not assigned (dummy variable)	42,100	—	—
**Ecological covariates - per 10% increase**			
% unemployed	—	1.082	1.056–1.109
% not graduated high school	—	1.026	1.020–1.031
% low income	—	0.959	0.951–0.967
**Population density – per IQR increase**			
Log population per km^2^ – Dissemination Area	—	0.996	0.991–1.000
**Aboriginal status**			
Not Aboriginal^§^	2,304,700	1.000	—
Aboriginal	143,700	1.704	1.673–1.736

^§^Reference category. ^†^Hazard ratios stratified by age (5 year categories) and sex. ^‡^Based on first year of postal code data included or imputed for each respondent.

**Table 2:** Descriptive statistics for PM_2.5_ (μg/m^3^), O_3_ (ppb), NO_2_ (ppb), and O_x_ (ppb) for all person-years in the cohort.

Pollutant	Percentile					Mean	Minimum	Maximum
	5^th^	25^th^	50^th^	75^th^	95^th^			
PM_2.5_	3.51	5.37	7.12	9.07	11.97	7.37	<1	20.00
O_3_	27.61	33.66	38.11	42.63	50.51	38.29	<1	60.46
NO_2_	3.36	6.44	10.31	15.10	24.52	11.47	<1	64.78
O_x_	20.41	25.42	29.57	32.98	37.76	29.17	<1	49.30

### Single Pollutant Models

**Figure 1:**
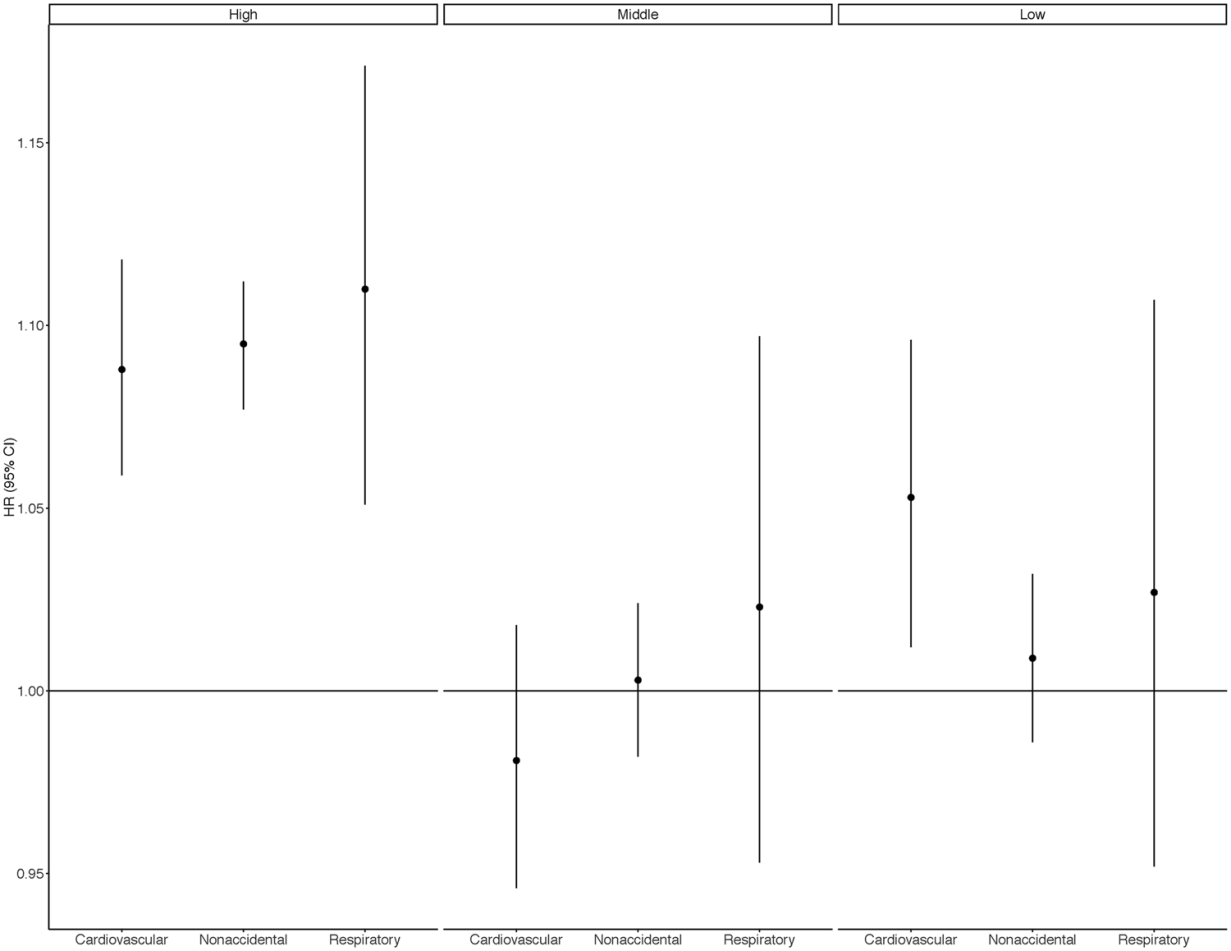
Hazard Ratios (95% CI) for relationships between PM_2.5_ and mortality (nonaccidental, cardiovascular, and respiratory) across tertiles of O_x_ in CanCHEC 2001. Hazard ratios reflect a 3.858 µg/m^3^ change in PM_2.5_.

**Figure 2:**
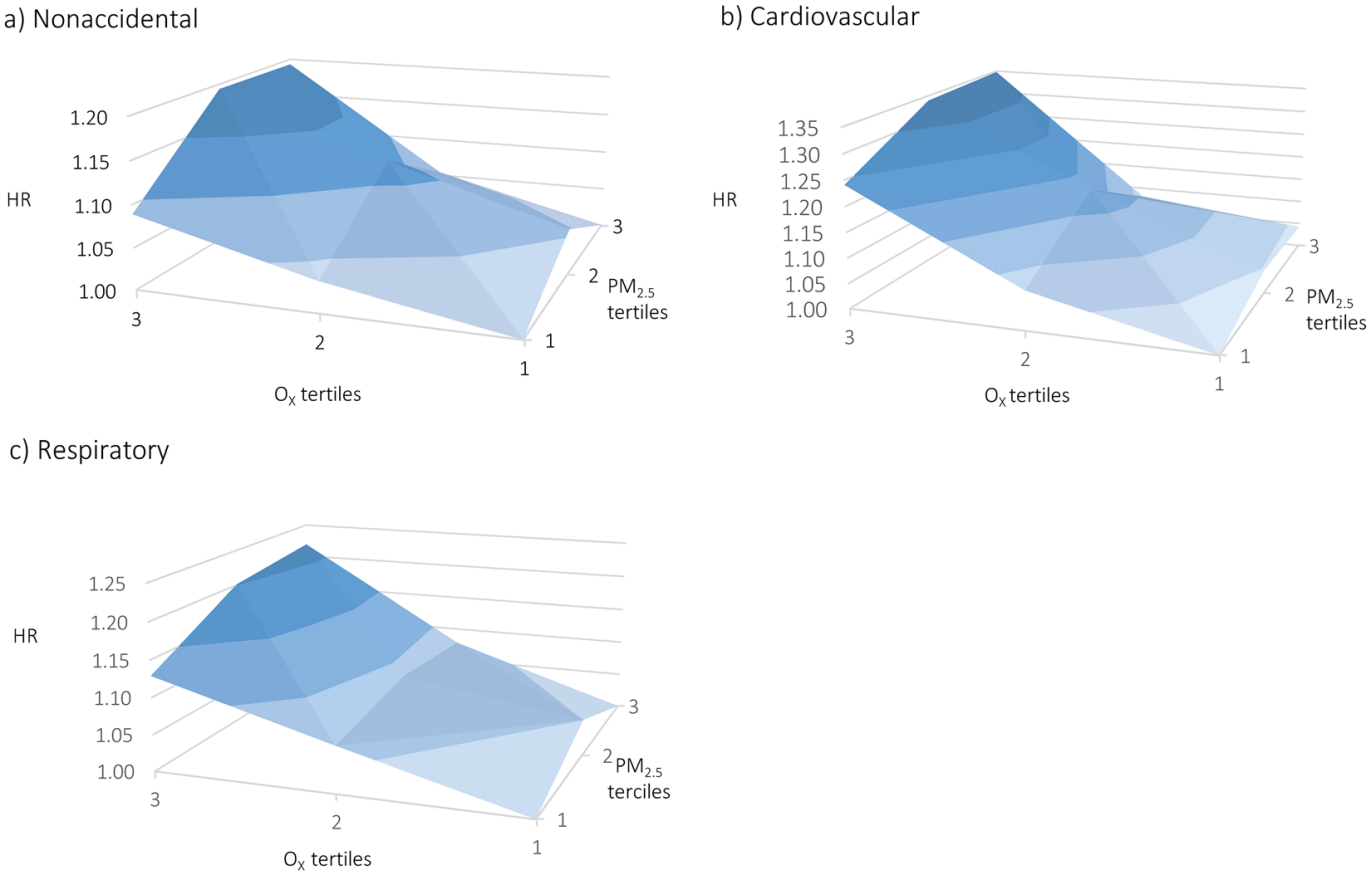
Three-dimensional plots of hazard ratios (HR) for (**a**) nonaccidental, (**b**) cardiovascular, and (**c**) respiratory mortality across tertiles of PM_2.5_ and O_x_ in CanCHEC 2001. Mean values across PM_2.5_ tertiles are 4.58 µg/m^3^ (1), 7.18 µg/m^3^ (2), and 10.35 µg/m^3^ (3). Mean values across O_x_ tertiles are 23.0 ppb (1), 29.2 (2), and 35.3 ppb (3).

All four pollutants (PM_2.5_, NO_2_, O_3_, and O_x_) were associated with increased nonaccidental, cardiovascular, and respiratory mortality in single pollutant models including linear terms for each pollutant (Table 3). Hazard ratios for O_x_ were generally higher than for O_3_ or NO_2_ individually except for respiratory mortality which was similarto that for NO_2_. For PM_2.5_, analyses across tertiles of O_x_ suggested consistently stronger associations in areas with higher oxidant gas concentrations for all three mortality outcomes (Fig. 1 and Supplemental Table 1). Similar trends were less apparent across tertiles of NO_2_ or O_3_ individually, particularly for O_3_; however, risks of nonaccidental and respiratory mortality did increase across tertiles of NO_2_ (Supplemental Table 2). The three-dimensional plots shown in Fig. 2 illustrate interactions across tertiles of PM_2.5_ and O_x_ for nonaccidental, cardiovascular, and respiratory mortality: for all three outcomes PM_2.5_-mortality associations were strongest in the highest tertile of O_x_.

**Table 3:** Hazard Ratios (95% CI) for relationships between nonaccidental, cardiovascular, and respiratory mortality and PM_2.5_, NO_2_, O_3_, and O_x_ in CanCHEC 2001.

COD	Deaths	Air Pollutants			
		PM_2.5_	O_3_	NO_2_	O_x_
		HR (95% CI)	HR (95% CI)	HR (95% CI)	HR (95% CI)
NAC	233,340	1.073 (1.062–1.083)	1.061 (1.051–1.070)	1.055 (1.046–1.064)	1.088 (1.077–1.099)
CV	77,000	1.107 (1.089–1.127)	1.170 (1.152–1.188)	1.045 (1.030–1.060)	1.198 (1.177–1.219)
RESP	21,100	1.089 (1.053–1.126)	1.043 (1.012–1.074)	1.091 (1.061–1.122)	1.086 (1.050–1.123)

COD, cause of death; NAC, non-accidental; CV, cardiovascular; RESP, respiratory. HRs reflect a 3.858 µg/m^3^ change in PM_2.5_, an 8.111 ppb change in NO_2_, a 10.503 ppb change in O_3_, and an 8.760 ppb change in O_x_.

### Two Pollutant Models (PM_2.5_ and O_x_)

**Figure 3:**
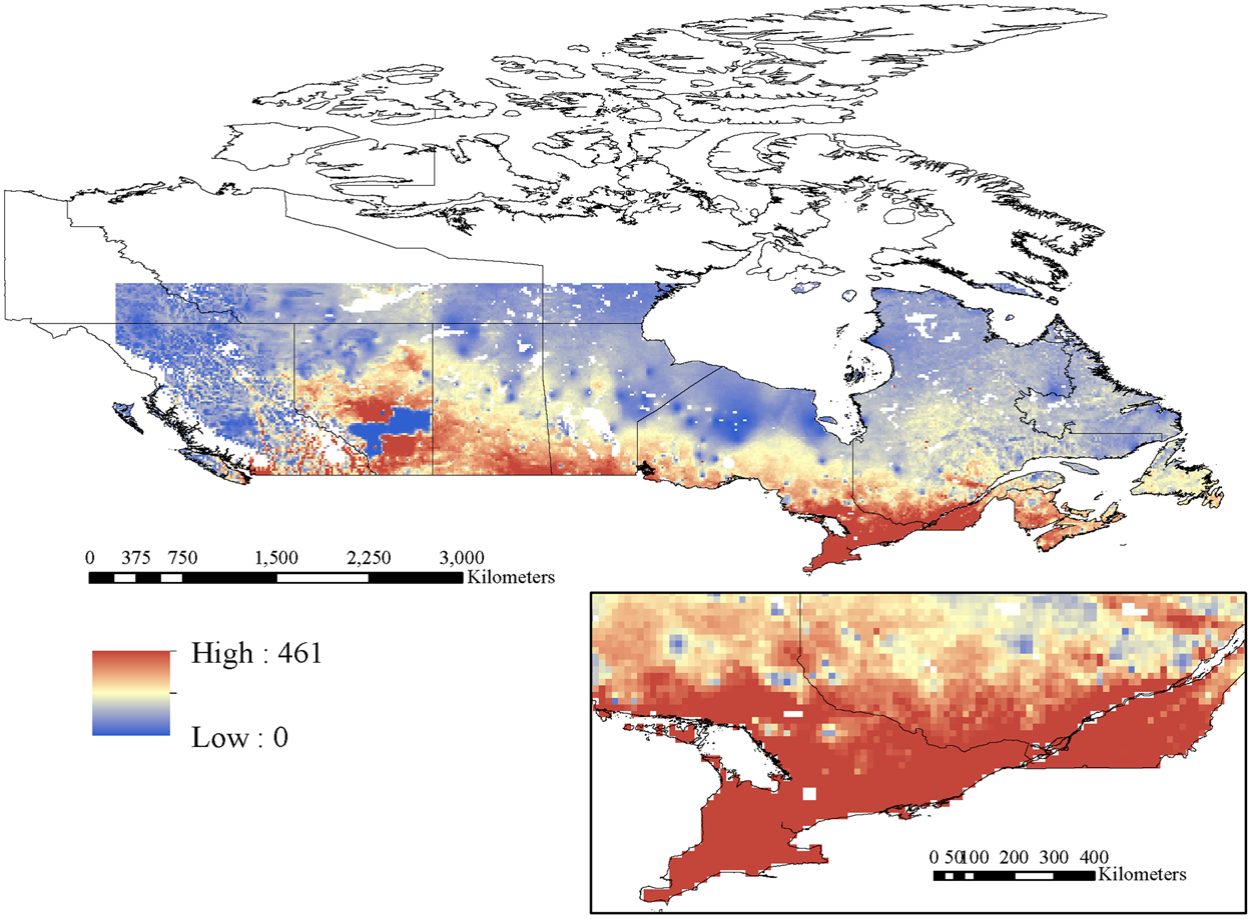
Spatial distribution of PM_2.5_*O_x_ (ppb*μg/m^3^) across Canada. Map created in ArcGIS Desktop 10.0. ESRI, Redlands, CA (http://desktop.arcgis.com/en/arcmap/).

When two-pollutant models were examined including linear terms for PM_2.5_ and O_x_, hazard ratios for PM_2.5_ decreased for all three mortality outcomes but remained elevated. The largest change occurred for the hazard ratio between PM_2.5_ and cardiovascular mortality which decreased from 1.107 (95% CI: 1.089, 1.127) in the single pollutant model to 1.024 (95% CI: 1.004, 1.044) in the two-pollutant model. The best fitting model for cardiovascular mortality included PM_2.5_, O_x_, and their interaction term (Table 4). For nonaccidental and respiratory mortality, model fit was similar between two pollutant models (i.e. containing PM_2.5_ and O_x_) and models including both pollutants and their interaction term. The spatial distribution of PM_2.5_*O_x_ is presented in Fig. 3 and highlights that the highest combined concentrations of PM_2.5_ and O_x_ occur in the most populated areas of Ontario and Quebec and along the southern border of Canada.

**Table 4:** Potentiation of the relationship between PM_2.5_ and mortality (nonaccidental, cardiovascular and respiratory mortality) by O_x_ in CanCHEC 2001.

Model and Cause of Death	−2(LL)	Pollutant					
		O_x_		PM_2.5_		PM_2.5_*O_x_	
		HR	95% CI	HR	95% CI	HR	95% CI
***PM***_***2.5***_							
Nonaccidental	3809180			1.073	1.062–1.083		
Cardiovascular	1227621			1.107	1.089–1.127		
Respiratory	330773			1.089	1.053–1.126		
***PM***_***2.5***_ + ***O***_***x***_							
Nonaccidental	3809055	1.067	1.055–1.080	1.041	1.029–1.052		
Cardiovascular	1227350	1.184	1.160–1.208	1.024	1.004–1.044		
Respiratory	330765	1.055	1.016–1.096	1.062	1.023–1.103		
***PM***_2.5_*O_***x***_							
Nonaccidental	3809092					1.054	1.047–1.060
Cardiovascular	1227481					1.094	1.082–1.105
Respiratory	330766					1.061	1.040–1.083
***PM***_***2.5***_ + ***PM***_2.5_*O_***x***_							
Nonaccidental	3809075			0.947	0.923–0.972	1.087	1.070–1.104
Cardiovascular	1227388			0.800	0.764–0.837	1.242	1.208–1.278
Respiratory	330766			0.979	0.897–1.067	1.075	1.019–1.134
***PM***_***2.5***_ + ***O***_***x***_ + ***PM***_2.5_*O_***x***_							
Nonaccidental	3809054	1.056	1.032–1.081	1.019	0.978–1.061	1.026	0.978–1.077
Cardiovascular	1227346	1.141	1.096–1.188	0.954	0.889–1.024	1.091	1.004, 1.187
Respiratory	330765	1.039	0.962–1.122	1.030	0.900–1.179	1.039	0.885–1.220

Hazard ratios for O_x_, PM_2.5_ and PM_2.5_*O_x_ reflect changes of 8.76 ppb, 3.858 µg/m^3^ and 100 ppb*µg/m^3^ respectively.

### Joint Non-linear Models for PM_2.5_ and O_x_

**Figure 4:**
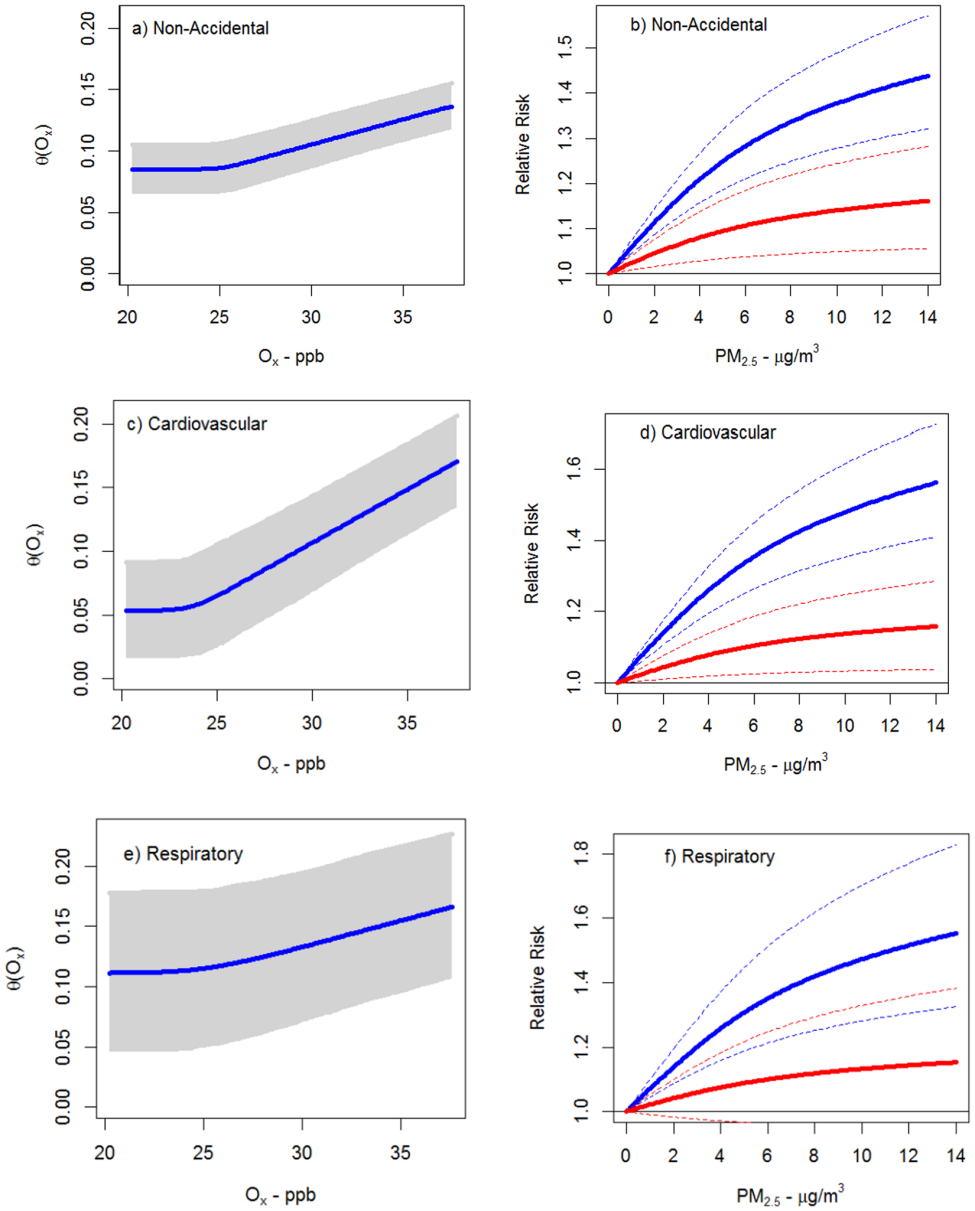
Predicted values (solid blue line) of $\hat{\theta }(O{}_{x})=\hat{\eta }+\hat{\lambda }{({O}_{x}-\omega )}_{+}$ by cause of death with uncertainty bounds (shaped gray area) (panels a, c, and e). Predicted values of $R(P{M}_{2.5})={(1+P{M}_{2.5})}^{\hat{\theta }({O}_{x})/(1+\exp (-z/2))}$ for O_x_ = 37.60 ppb (solid blue line) and O_x_ = 20.26 ppb (solid red line) with uncertainty intervals (dashed lines) (panels b, d, f).

Effect modification by O_x_ was also apparent when we examined non-linear model forms for PM_2.5_ -mortality associations (Fig. 4). Specifically, the shapes of associations between O_x_ and *θ* (the parameter describing the magnitude of association between PM_2.5_ and mortality) were similar for all three causes of death (Fig. 4, panels a, c, and e) with threshold concentrations of 23.71 ppb, 25.08 ppb, and 25.13 ppb for cardiovascular, respiratory and nonaccidental mortality, respectively. The rate of change in *θ* per ppb of O_x_ above the threshold was greatest for cardiovascular mortality and similar for both non-accidental and respiratory mortality (*λ* values in the Supplemental Methods). Figure 4 (panels b, d, and e) illustrates non-linear concentration response relationships for PM_2.5_ and nonaccidental, cardiovascular, and respiratory mortality at O_x_ concentrations of 20.26 ppb (red solid line) and 37.6 ppb (blue solid line) which represent mean concentrations in the first and tenth deciles respectively of the O_x_ distribution. This Figure clearly illustrates considerable variations in PM_2.5_-mortality associations at these two O_x_ concentrations.

**Figure 5:**
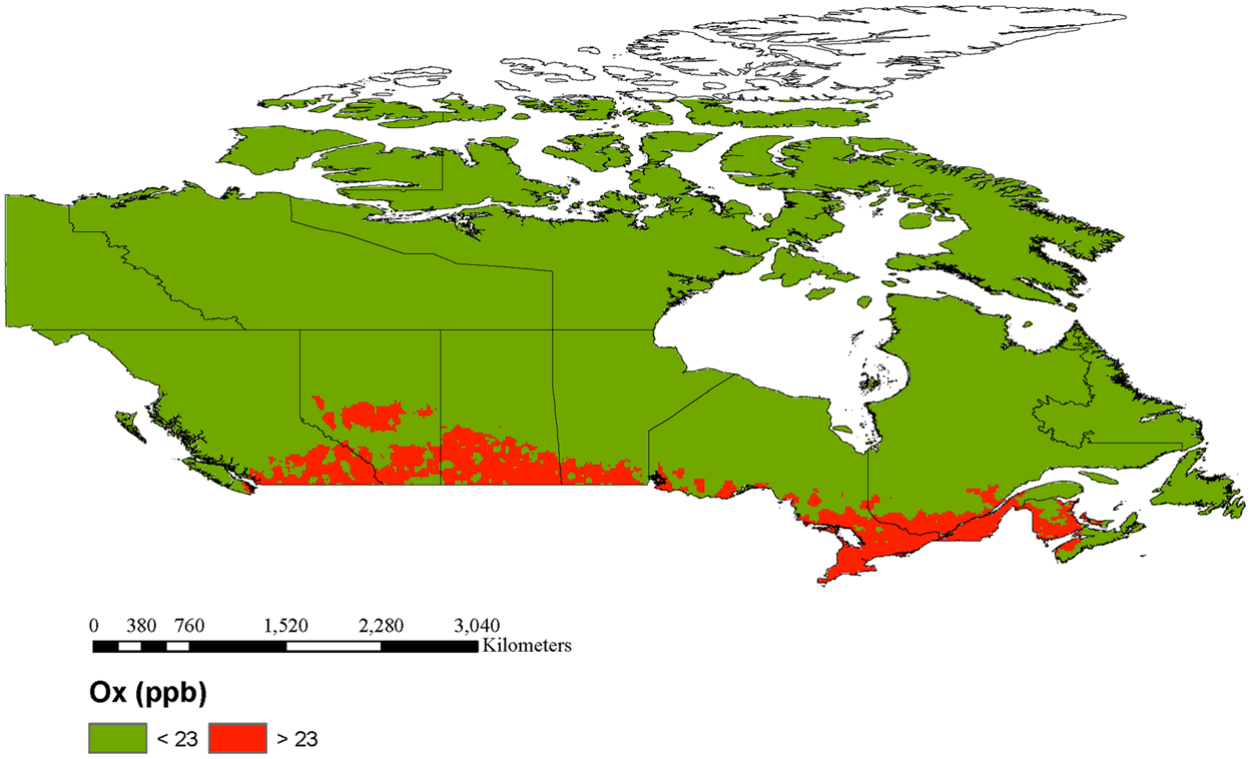
Regions of Canada with estimated O_x_ concentrations above 23.0 ppb. Reductions in O_x_ concentrations in these areas are expected to decrease the chronic health risks of PM_2.5_ even if mass concentrations remain unchanged. Map created in ArcGIS Desktop 10.0. ESRI, Redlands, CA (http://desktop.arcgis.com/en/arcmap/).

Finally, Fig. 5 highlights areas of Canada with estimated O_x_ concentrations above 23 ppb. Based on the evidence above, reductions in O_x_ concentrations in these regions are expected to reduce the chronic health effects of PM_2.5_ even if mass concentrations remain unchanged.

## Discussion

To our knowledge, this is the first large population-based cohort study to evaluate how oxidant gases may modify the chronic health risks of PM_2.5_. In general, our findings suggest that oxidant gases act to enhance PM_2.5_-mortality associations and that this effect modification occurs at O_x_ concentrations above approximately 23 ppb. This finding should be confirmed in future cohort studies as to our knowledge this study is the first to report such a relationship. If confirmed, these findings are important as they indicate that in some areas reductions in O_x_ concentrations may have the added benefit of reducing the public health impacts of PM_2.5_ even if mass concentrations remain unchanged.

While other studies have not examined the impact of oxidant gases on the chronic health effects of PM_2.5_, we previously reported that O_x_ modified the association between short-term changes in ambient PM_2.5_ and the risk of emergency room visits for myocardial infarction^8^. In addition, others have reported associations between daily variations in O_x_ and mortality in London, England^9^. One possibility is that spatial variations in O_x_ concentrations are reasonable surrogates for the presence/absence of harmful air pollution mixtures and/or sources that are more relevant to health and thus PM_2.5_ in these regions tends to be more harmful. However, other biological mechanisms may also explain our observation of stronger PM_2.5_-mortality associations in regions with higher O_x_ concentrations. First, ozone rapidly saturates the lung epithelial lining fluid where it degrades surfactants and depletes anti-oxidants; thus, oxidant gases may reduce our natural defense against reactive oxygen species generated in response to PM_2.5_ components including transition metals, polycyclic aromatic hydrocarbons, or quinones^3,10^. In addition, ozone increases the permeability of the lung epithelium barrier^4–7^ and thus may facilitate the translocation of particles (or inflammatory proteins) from the lungs directly into the systemic circulation. Moreover, endogenous sources of oxidative stress may also pay an important role as NO_2_ has been shown to induce the release of reactive oxygen species from alveolar macrophages^11^. Alternatively, photochemical aging of PM_2.5_ has been shown to increase the oxidative potential of particles themselves^12,13^; therefore, this process may also contribute to our observation of stronger PM_2.5_-mortality associations in regions with higher oxidant gas concentrations as photochemical oxidation would be greater in these areas.

One interesting finding in our investigation was that the rate of change in *θ* (the parameter describing the magnitude of association between PM_2.5_ and mortality) per ppb of O_x_ above the threshold was greater for cardiovascular mortality than respiratory mortality. One explanation for this finding may be that PM_2.5_ components and/or inflammatory mediators must first reach the systemic circulation from the lung in order to contribute to cardiovascular morbidity. If this process happens more quickly/efficiently at higher O_x_ concentrations (perhaps owing to increased lung permeability as noted above) this may explain the larger slope for *θ* in cardiovascular mortality. While we could not directly evaluate this question in the current study, future studies should aim to replicate this result as it may contribute to our understanding of how particulate air pollution influences cardiovascular morbidity/mortality.

This study had several important advantages including a large population-based sample of Canadians, exposure information for multiple air pollutants updated for residential mobility, detailed non-linear concentration-response modeling, and individual-level information on many important socio-economic factors. However, we cannot rule out potential confounding by unmeasured variables including smoking and obesity. In addition, as in all large scale epidemiological studies, exposure measurement error is a challenge and likely impacted our results. Specifically, the spatial scale of O_3_ estimates was large and greater resolution is needed to refine or assessment of the spatial distribution of O_x_ (and PM_2.5_*O_x_) and associated health impacts. In general, measurement error for all air pollutants likely contained components of both Classical (i.e. grid-cell mean values distributed around true long-term ambient concentrations) and Berkson-type error (i.e. true individual-level exposures distributed around grid-cell mean values). Classical type error would tend to bias risk estimates toward the null whereas Berkson type error would tend to increase uncertainty in our estimates (i.e. wider confidence intervals). In either case, this error is not a likely explanation for our observed pattern of stronger PM_2.5_-mortaltiy associations in areas with higher O_x_ concentrations. Moreover, it is important to note that there is no clear consensus on the optimal spatial scale at which to evaluate long-term exposures to outdoor air pollution. Currently, the same spatial scale is applied to all cohort members but a more optimal approach may be to tailor the spatial scale of exposure assessment to match the size of individual-level activity spaces based on individual-level covariates such as age, sex, or socioeconomic status (e.g. older people may spend more time at home and thus a smaller spatial scale may be more appropriate for older cohort members). In some cases, larger spatial scales may be more appropriate if cohort members are highly mobile within their region and thus larger grid sizes are not necessarily a limitation. Nevertheless, more work is needed to refine air pollution exposure assessment in large population-based studies and our future work will aim to address several of these issues.

In summary, the results of our cohort study suggest that oxidant gases may act to enhance the chronic health impacts of PM_2.5_. If confirmed, our findings may provide additional flexibility for regulators/risk managers in reducing the overall public health impacts of PM_2.5_. In particular, there may be important co-benefits to reducing O_x_ as reductions in oxidant gas concentrations may also reduce the chronic health impacts of PM_2.5_ even if mass concentrations remain unchanged. The choice of which pollutant(s) to target in a given area will likely be situation-specific; however, knowledge of how oxidant gases and PM_2.5_ may interact to cause adverse health impacts could improve the efficiency of risk management activities and ultimately public health.

## Methods

### Study Population

This study uses the 2001 Canadian Census Health and Environment Cohort (CanCHEC) described in Pinault *et al*.^14^. Briefly, the 2001 Census long-form questionnaire was distributed to approximately 20% of Canadian households including 4,500,200 Census respondents aged 19 years or older who did not reside in institutions and who lived in Canada^15^. Of these, 78.6% (n = 3,537,500) were linked to income tax files to obtain annual postal code histories and Social Insurance Numbers through standard deterministic and probabilistic linkage techniques^16^. These subjects were then deterministically linked to the Amalgamated Mortality Database using Social Insurance Numbers. All deaths that occurred between census day (May 15, 2001) and December 31, 2011 were eligible for linkage.

Respondents who were not assigned air pollution estimates due to living outside the boundaries of air pollution models were excluded (n = 86,100) as were those less than 25 years of age or older than 90 years (n = 319,900 additional persons excluded). All immigrants were also excluded from the cohort (n = 683,100) since their previous air pollution exposures were unknown. The final analytical sample was 2,448,500 respondents: sample sizes are rounded to the nearest hundred for confidentiality.

Postal code histories obtained from tax records were used to geocode respondent addresses. Statistics Canada’s Postal Code Conversion File plus (PCCF+) v.6c uses a population-weighted random allocation algorithm to assign geographic coordinates to postal codes based on centroids of different scales of Census geography^17^. The gaps in postal code reporting (approximately 18% of all person-years) were imputed using a probabilistic imputation method developed at Statistics Canada, which assigned postal code common characters from postal codes provided before and after the gap in reporting^18^. The method included a non-null probability that missing postal codes would be different from adjacent years, and the probability of non-matches increased with increasing gap length. In cases where postal codes were not imputed using any common characters, the national mean exposure estimate was assigned. In a validation exercise with the 1991 CanCHEC, when 5% of postal codes were randomly deleted and then imputed using the program, 4.2% of imputed postal codes had an absolute PM_2.5_ difference greater than 0.1 μg/m^3^ ^18^. In general, 2001 CanCHEC members were more likely to be married or common-law, have higher income or higher educational attainment, or be employed than were the general Canadian population^14^.

### Exposure Assessment

Cohort members were assigned air pollution exposure estimates for PM_2.5_, NO_2_, and O_3_ using models that have been previously described. Specifically, PM_2.5_ exposures were derived from a surface model that combines information for total column aerosol optical depth retrievals from the Moderate Resolution Imaging Spectroradiometer (MODIS) with near-surface PM_2.5_ emissions estimated from the GEOS-Chem chemical transport model^19^. Yearly (2012 to 2014) average surface layers of PM_2.5_ (at 1 km^2^ resolution) were obtained by applying geographically weighted regression, and extended back in time to 1998 by applying inter-annual variation from a published model^20^. Within North America, mean PM_2.5_ estimates were strongly correlated with ground level measurements (R^2^ = 0.82, slope = 0.97, n = 1440)^19^. Estimates of PM_2.5_ that were greater than 20 μg/m^3^ were excluded from the analysis because they likely represented inaccurate satellite retrievals.

Outdoor NO_2_ concentrations were estimated using the 2006 annual mean from a national land use regression model that used National Air Pollution Surveillance (NAPS) fixed-site monitoring data combined with satellite NO_2_ estimates, road length within 10 km, industrial land use areas within various buffers, and mean summer rainfall^21^. Ground-level NO_2_ estimates were derived using GEOS-Chem from satellite tropospheric NO_2_ columns^22^. During validation, the model explained 73% of the variance in 2006 NAPS estimates. Local variation in NO_2_ was captured by applying kernel density measures of highways and major roads as a multiplier to the model^21^.

A surface for average daily 8-hr maximum O_3_ concentration was generated for the months of May to October for the period of 2002 to 2009 using an air pollution-specific interpolation technique to generate a 21 km^2^ grid^2,23^. This method incorporates modeled O_3_ from the Canadian Hemispheric Regional Ozone and NOx system (CHRONOS) air quality forecast model^24^ with observations from Canada and the United States.

All NO_2_ and ozone data were year-adjusted using ground-based time-series measurements from 24 Census Divisions (CD)s between 1981 and 2012. The time series for NO_2_ and O_3_ were derived from NAPS daily average concentrations (if at least 18 hourly concentrations were recorded in a day), and averaged for CDs with more than one monitoring station^25^. Missing time-series data were imputed using an interpolation algorithm that combines classical prediction techniques and the phase-and frequency-fitting tools via the multi-taper method using the R package *tsinterp* ^26^. For each CD time-series, a cubic spline was fitted to model the association between year and air pollutant concentration. Then, the ratio between the year of the original modeled data (i.e., NO_2_: 2006, O_3_: mean of 2002 to 2009) and all years of follow-up was determined. For each year of follow-up, residence locations for cohort members were matched to the closest CD in Geographic Information Systems (ArcGIS v.10, ESRI 2010), and the corresponding time adjustment ratio was used to adjust data for annual differences in concentration. All air pollution exposures were assigned using 3-year moving averages with a 1-year lag time (and updated for residential mobility).

Finally, the combined oxidant capacity (O_x_) of O_3_ and NO_2_ at each residential location was calculated as a weighted average with weights equivalent to their respective redox potentials (i.e. O_x_ = [(1.07 × NO_2_) + (2.075 × O_3_)]/3.145)^27^.

### Statistical Analyses

Standard Cox proportional hazards models were used to estimate hazard ratios (HR) and 95% confidence intervals (95% CI) describing the relationship between outdoor air pollutants and nonaccidental (ICD-10: A to R), cardiovascular (ICD-10: I10-69 and E10-E14 (diabetes)), and respiratory mortality (ICD-10: J00-J99). All models were stratified by age (5-year categories), sex, airshed, and population centre size, and adjusted for individual-level covariates including visible minority status, Aboriginal status, marital status, educational attainment, income quintile, and labour force status. In addition, several neighbourhood-level covariates were included in the models including percent unemployed (aged 25 and older), percent not graduated from high school (aged 25 and older), and percent low income status within census divisions as well as population density (per km^2^) within dissemination areas (Table 1). All hazard ratios for individual pollutants are expressed for intervals equivalent to the difference between the mean and the 5^th^ percentile of each pollutant. Hazard ratios for the product of PM_2.5_ and O_x_ are expressed per 100 ppb*µg/m^3^.

A series of models were examined to evaluate how oxidant gases may modify the relationship between PM_2.5_ and mortality. First, single pollutant models were examined using linear terms for PM_2.5_, NO_2_, O_3_, and O_x_ (as defined above). Hazard ratios for PM_2.5_ were then calculated across tertiles of O_x_ to evaluate trends in PM_2.5_-mortality associations at increasing concentrations of oxidant gases; the entire range of PM_2.5_ concentrations (i.e. 1–20 µg/m^3^) was available within each tertile of O_x_. Three-dimensional plots were also examined for hazard ratios across tertiles of O_x_ and PM_2.5_.

Next, two pollutant models including both PM_2.5_ and O_x_ were examined to evaluate potential confounding by O_x_ in PM_2.5_-mortality associations followed by: 1) models including only the product of PM_2.5_ and O_x_ (i.e. PM_2.5_*O_x_); 2) models including PM_2.5_ and the product of PM_2.5_ and O_x_ (i.e. PM_2.5_ + PM_2.5_*O_x_); and 3) models including PM_2.5_, O_x_, and product of PM_2.5_ and O_x_ (i.e. PM_2.5_ + O_x_ + PM_2.5_*O_x_). Our primary aim in evaluating this series of models was to examine how O_x_ may modify the relationship between PM_2.5_ and mortality using linear terms for individual pollutants both excluding and including the main effect of O_x_. Following the above analyses, we considered non-linear model forms to characterize potential effect modification due to O_x_ on PM_2.5_-mortality associations as outlined below.

The traditional relative risk function used to relate PM_2.5_ concentrations to mortality is: $R(P{M}_{2.5})=$ $\exp \{\theta \times P{M}_{2.5}\}$ with *θ* representing the logarithm of the relative risk for a unit change in PM_2.5_, assuming a linear relationship between PM_2.5_ and the logarithm of the relative risk. This model has been extended to include non-linear model specifications of the form: $R(P{M}_{2.5})=\exp \{\theta \times T(P{M}_{2.5})\}$,where *T*(*PM*_2.5_) is a monotonic transformation of PM_2.5_^28,29^. Here, *θ* represents the logarithm of the relative risk for a unit change in *T*(*PM*_2.5_). Setting $T(P{M}_{2.5})=\mathrm{log}(\Im (P{M}_{2.5}))/1+\exp (-(P{M}_{2.5}-\mu )/\pi )$, where $\Im (z)={e}^{z}$ or $\Im (z)=1+z$, specifies a family of shapes taking supra-linear, near-linear, sub-linear and sigmodal forms depending on $(\Im ,\mu ,\pi )$.

In this study, we examined how mortality risks due to PM_2.5_ exposures may be affected by co-occurring exposure to O_x_. To do this, we introduced the notion that the parameter *θ* can vary with concentrations of O_x_ by defining a joint relative risk function of the form: $R(P{M}_{2.5},{O}_{x})=\exp \{\theta ({O}_{x})\times T(P{M}_{2.5})\}$. We additionally postulated that there exists a concentration of O_x_, *ω*, below which, O_x_ does not modify the risk of death due to PM_2.5_ exposure and above that concentration there exists a linear association. That is: $\theta ({O}_{x})=\eta +\lambda \times {({O}_{x}-\omega )}_{+}$, such that ${({O}_{x}-\omega )}_{+}=0$ if *O*_*x*_ < *ω* and ${({O}_{x}-\omega )}_{+}=({O}_{x}-\omega )$ if ${O}_{x}\ge \omega$, for concentration *ω*. It is important to note that this model does not directly evaluate the impact of O_x_ on mortality; rather, it redistributes PM_2.5_ risk according to concentrations of O_x_. Methods to estimate the unknown parameters and their uncertainty are presented in the Supplemental Material.

All statistical analyses were conducted using R version 3.2.4 and SAS version 9.3. Map Figures were created in ArcGIS 10.0 (ESRI, 2010). NO_2_ and O_3_ surfaces were created using interpolation (i.e. inverse distance weighted) from nationally representative NO_2_ and O_3_ point estimates, and raster algebra was used to calculate O_X_ and the product of O_X_ and PM_2.5_ using published PM_2.5_ surfaces^19^. All data are available through the Statistics Canada Research Data Centers located across Canada conditional on the necessary institutional approvals from Statistics Canada including security screening.

### Institutional Approvals

The Canadian Census Health and Environment Cohort (CanCHEC) was approved by the Statistics Canada Policy Committee (reference number 012-2001) after consultation with the Statistics Canada Confidentiality and Legislation Committee, Data Access and Control Services Division, and the Federal Privacy Commissioner. This approval is equivalent to that of standard research ethics boards.

## Electronic supplementary material

Supplemental Material
