# Supplementary material for: Impact of Oxidant Gases on the Relationship between Outdoor Fine Particulate Air Pollution and Nonaccidental, Cardiovascular, and Respiratory Mortality
Source: Sci Rep. 2017 Nov 27;7:16401. doi: 10.1038/s41598-017-16770-y (PMC5703979; doi:10.1038/s41598-017-16770-y)

SUPPLEMENTAL MATERIAL

Impact of Oxidant Gases on the Relationship between Outdoor Fine Particulate Air Pollution and Nonaccidental, Cardiovascular, and Respiratory Mortality

Scott Weichenthal[1,2*], Lauren L Pinault[3], Richard T Burnett[4]

[1]Department of Epidemiology, Biostatistics, and Occupational Health, McGill University, Montreal, QC, Canada
[2]Air Health Effects Science Division, Health Canada, Ottawa, ON, Canada
[3]Health Analysis Division, Statistics Canada, Ottawa, ON, Canada
[4]Population Studies Division, Health Canada, Ottawa, ON, Canada

*Corresponding Author
Scott Weichenthal, PhD
Assistant Professor
Department of Epidemiology, Biostatistics, and Occupational Health
McGill University
1020 avenue des Pins Ouest
Montreal, QC H3A 1A2
Canada
Email: scott.weichenthal@mcgill.ca
Tel: (514) 398-1584

Supplemental Methods

Estimation of Parameters in the Joint O_x - $PM_{2.5}$ ***Relative Risk Model:*** The joint O_x - $PM_{2.5}$ relative risk model has the form

$$R(PM_{2.5}) = \exp\left\{\left(\hat{\eta} + \hat{\lambda}(O_x - \omega)_+\right) \times \log(\Im(PM_{2.5}))/(1 + \exp(-(PM_{2.5} - \mu)/\pi))\right\}.$$

The six unknown parameters cannot be directly estimated with the Cox proportional hazards model using standard computer software since our model is intrinsically non-linear in these parameters. Due to the large number of observations in the cohort (over 20 million person-years) computer intensive estimation methods such MCMC within a fully Bayesian framework are not practical. We therefore estimate the unknown parameters in the following 2-stage process. Our joint relative risk model assumes that the shape of the association between $PM_{2.5}$ and mortality is the same for all values of O_x and only the magnitude of the association is allowed to vary. We are thus not modelling a full joint relative risk model where the shape of the relative risk function can vary in an arbitrary manner with both pollutants. We are not aware of computer software that can model sure a joint function within the Cox proportion hazards framework that would be monotonically increasing in both pollutants.

Stage 1- estimation of $(\Im, \mu, \pi)$: Here we assume that θ is a constant, independent of O_x. We first postulate a relative risk model as only a function of $PM_{2.5}$ and not of O_x with the form: $R(z) = \Im(z)^{\psi(z)}$, where $\Im(z) = e^z$ or $\Im(z) = 1 + z$, and $\psi(z) = \theta/(1 + \exp(-(z - \mu)/(\tau \times r)))$ with r the range in the $PM_{2.5}$. The estimation of the unknown parameters has been described elsewhere in detail[1,2]. Briefly, the estimation method selects values of $(\Im, \mu, \tau)$ and estimates θ using standard computer software that fit the Cox proportional hazards model to the cohort survival

data[1]. We ran several Cox models with $\tau = 0.1$ or $\tau = 0.2$, $\Im(z) = e^z$ or $\Im(z) = 1 + z$, and μ taking concentrations of $PM_{2.5}$ at each 5 percentile value. Values of $(\Im, \mu, \pi)$ were selected based on minimizing the log-likelihood function. For all three causes of death our routine selected $\tau = 0.1$, $\Im(z) = 1 + z$, and $\mu = 0\mu g / m^3$, the minimum concentration. This resulted in the transformation: $T(PM_{2.5}) = \log(1 + PM_{2.5})/(1 + \exp(-PM_{2.5}/2))$, since $r = 20\mu g / m^3$ and thus $\tau \times r = 2$.

Stage 2 – estimation of (η, λ, ω): We fit a Cox model with two pollution variables. The first is $T(PM_{2.5})$ and the second is $(O_x - \omega)_+ \times T(PM_{2.5})$. We fit 10 of these models selecting values of ω as its decile means, resulting in 10 estimates of η and λ, and their estimated 2 by 2 covarince matrix, $\hat{V}$. We then select the value of ω, $\hat{\omega}$,that minimizes the -2 log-likelihood function as our best estimate, with the corresponding covariance matrix. We estimated the uncertainty in $\hat{\omega}$ by first approximating the -2 times the log-likelihood (-2LL) values by a quadric polynomial in O_x and defined a 95% confidence interval for $\hat{\omega}$ as those O_x values corresponding to the -2LL values within 3.84 units. We then estimated a standard deviation of a normal distribution, $\hat{\sigma}$, by dividing the length of the confidence interval by 2×1.96. Uncertainty in our predictions of $\hat{\theta}(O_x) = \hat{\eta} + \hat{\lambda}(O_x - \omega)_+$ are derived by bootstrap methods assuming multivariate normal distribution for $(\hat{\eta}, \hat{\lambda})$ with covariance matrix $\hat{V}$ and an independent univariate normal distribution for $\hat{\omega}$ with standard deviation $\hat{\sigma}$.

Parameter estimates of $\theta(O_x) = \eta + \lambda(O_x - \omega)_+$ and unique elements of covariance matrix, V , of estimates of $(\hat{\eta}, \hat{\lambda})$ with standard errors in parentheses.

Parameter[+]	*Cause of Death*		
	Non-Accidental	*Cardiovascular*	*Respiratory*
$\theta(O_x) = \eta + \lambda \times (O_x - \omega)_+$	$\eta = 0.08541(0.01005)$<br>$\lambda = 0.00408(0.00034)$<br>$\omega = 25.13(0.67)$	$\eta = 0.0536(0.01908)$<br>$\lambda = 0.00839(0.00061)$<br>$\omega = 23.71(0.98)$	$\eta = 0.1106(0.03356)$<br>$\lambda = 0.00441(0.00115)$<br>$\omega = 25.08(2.306)$
$V[1,1]$<br>$V[1,2]$<br>$V[2,2]$	1.01×10^{-4}<br>-1.69×10^{-6}<br>$1.16 * 10^{-7}$	3.64×10^{-4}<br>-6.38×10^{-6}<br>$3.75 * 10^{-7}$	11.26×10^{-4}<br>-18.91×10^{-6}<br>$13.12 * 10^{-7}$

Supplemental References

1. Nasari M, Szyszkowicz M, Chen H, Crouse D, Turner MC, Jerrett M, Pope CA III, Hubbell B, Fann N, Cohen A, Gapstur SM, Diver WR, Forouzanfar MH, Kim S-Y, Olives C, Krewski D, Burnett RT. (2015). A Class of Non-Linear Exposure-Response Models Suitable for Health Impact Assessment Applicable to Large Cohort Studies of Ambient Air Pollution. Air Quality, Atmosphere, and Health: DOI: 10.1007/s11869-016-0398-z.
2. Pinault LL, Weichenthal S, Crouse DL, Brauer M, Erickson A, van Donkelaar A, Martin RV, Hystad P, Chen H, Finès P, Brook JR, Tjepkema M, Burnett RT. Associations between fine particulate matter and mortality in the 2001 Canadian Census Health and Environment Cohort . Environmental Research, 159:406-415.

Supplemental Table 1. Hazard Ratios (95% CI) for relationships between $PM_{2.5}$ and mortality (nonaccidental, cardiovascular, and respiratory) across tertiles of O_x in CanCHEC 2001

Cause of death	Tertile of O_x		
	Low<br>HR (95% CI)	Middle<br>HR (95% CI)	High<br>HR (95% CI)
Nonaccidental	1.009<br>(0.986-1.032)	1.003<br>(0.982-1.024)	1.095<br>(1.077-1.112)
Cardiovascular	1.053<br>(1.012-1.096)	0.981<br>(0.946-1.018)	1.088<br>(1.059-1.118)
Respiratory	1.027<br>(0.952-1.107)	1.023<br>(0.953-1.097)	1.110<br>(1.051-1.171)

HRs reflect a 3.858 $\mu g/m^3$ change in $PM_{2.5}$.

Supplemental Table 2. Hazard Ratios (95% CI) for relationships between $PM_{2.5}$ and mortality (nonaccidental, cardiovascular, and respiratory) across tertiles of NO_2 and O_3 in CanCHEC 2001

Cause of death	Tertile of NO_2			Tertile of O_3		
	Low	Middle	High	Low	Middle	High
Nonaccidental	1.020 (0.999-1.041)	1.052 (1.033-1.072)	1.065 (1.044-1.087)	1.053 (1.031-1.076)	1.039 (1.019-1.060)	1.067 (1.051-1.083)
Cardiovascular	1.088 (1.050-1.128)	1.094 (1.059-1.129)	1.089 (1.051-1.128)	1.090 (1.050-1.131)	1.026 (0.990-1.063)	1.081 (1.054-1.110)
Respiratory	1.003 (0.936-1.075)	1.016 (0.955-1.082)	1.124 (1.047-1.205)	1.085 (1.011-1.164)	1.036 (0.968-1.109)	1.085 (1.030-1.142)

HRs reflect a 3.858 $\mu g/m^3$ change in $PM_{2.5;}$ Tertiles: NO_2 (Low: <7.66 ppb; Middle: 7.66-12.95 ppb; High: >12.95 ppb); O_3 (Low: <35.29 ppb; Middle: 35.29-40.43 ppb; High: >40.43 pp

Supplemental Figure S1. Spatial variations in ambient NO_2 across Canada. Map created in ArcGIS Desktop 10.0. ESRI, Redlands, CA (http://desktop.arcgis.com/en/arcmap/).

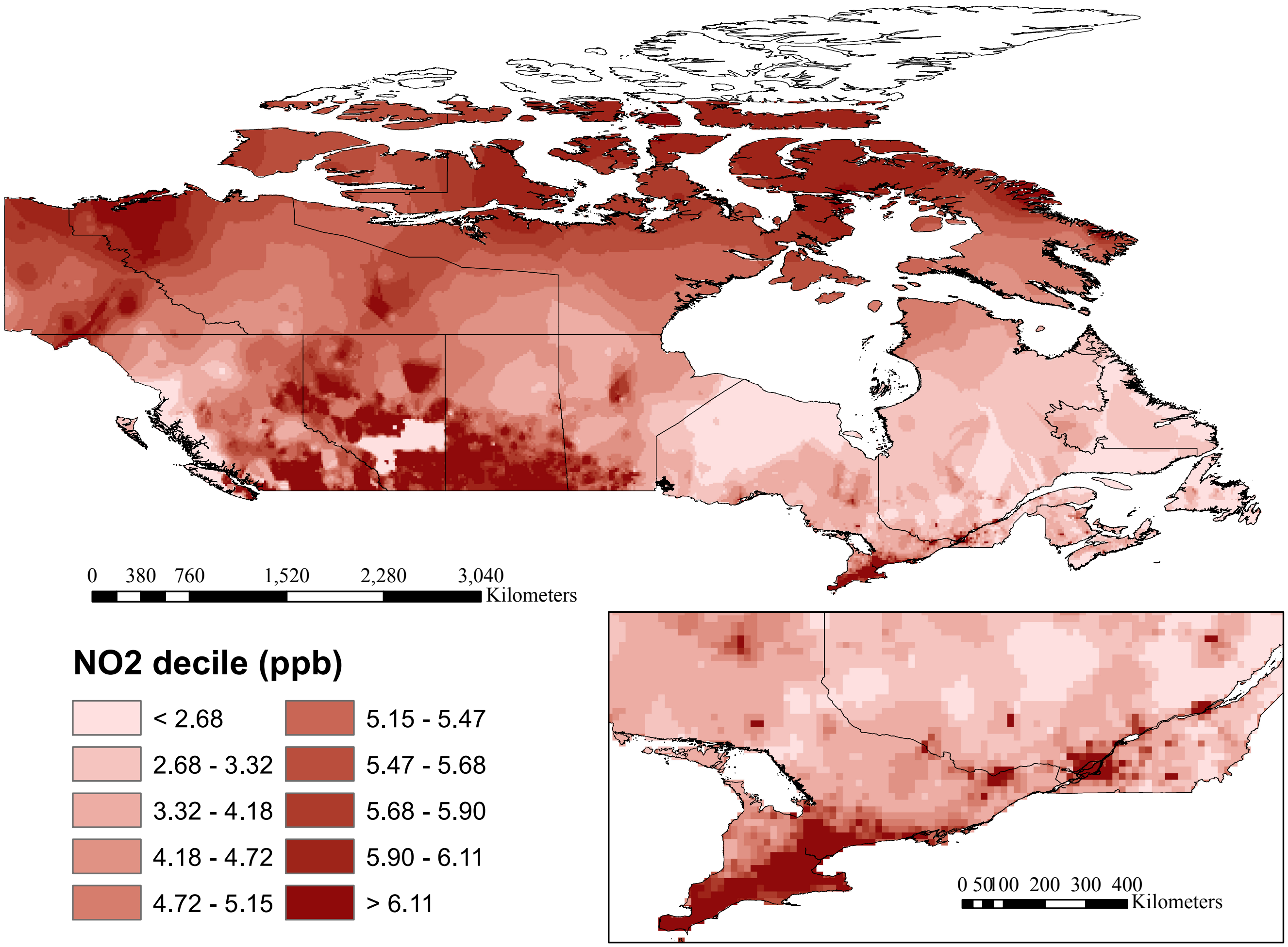

Supplemental Figure S2. Spatial variations in ambient O_3 across Canada. Map created in ArcGIS Desktop 10.0. ESRI, Redlands, CA (http://desktop.arcgis.com/en/arcmap/).

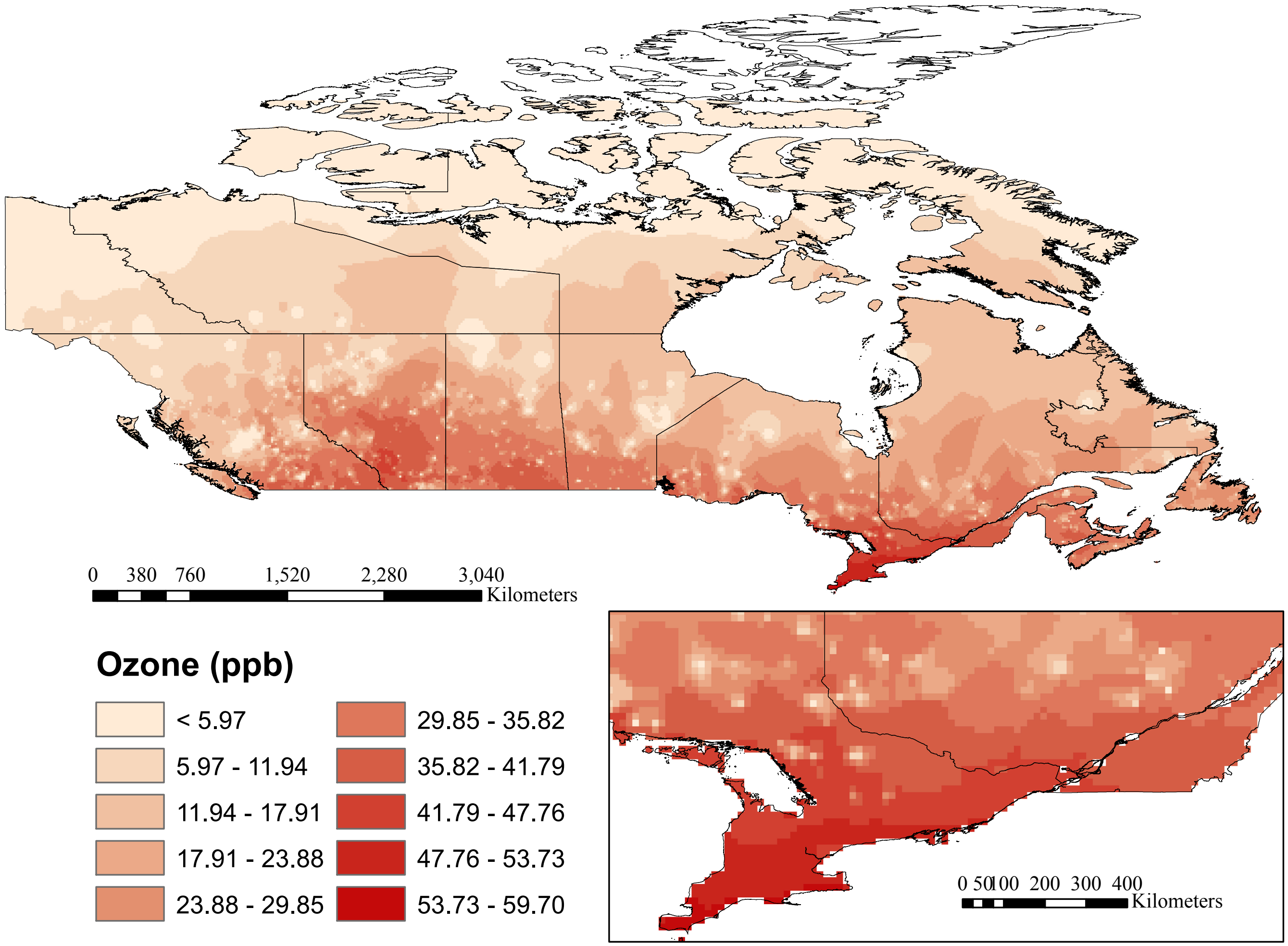

Supplemental Figure S3. Spatial variations in ambient $PM_{2.5}$ across Canada. Map created in ArcGIS Desktop 10.0. ESRI, Redlands, CA (http://desktop.arcgis.com/en/arcmap/).

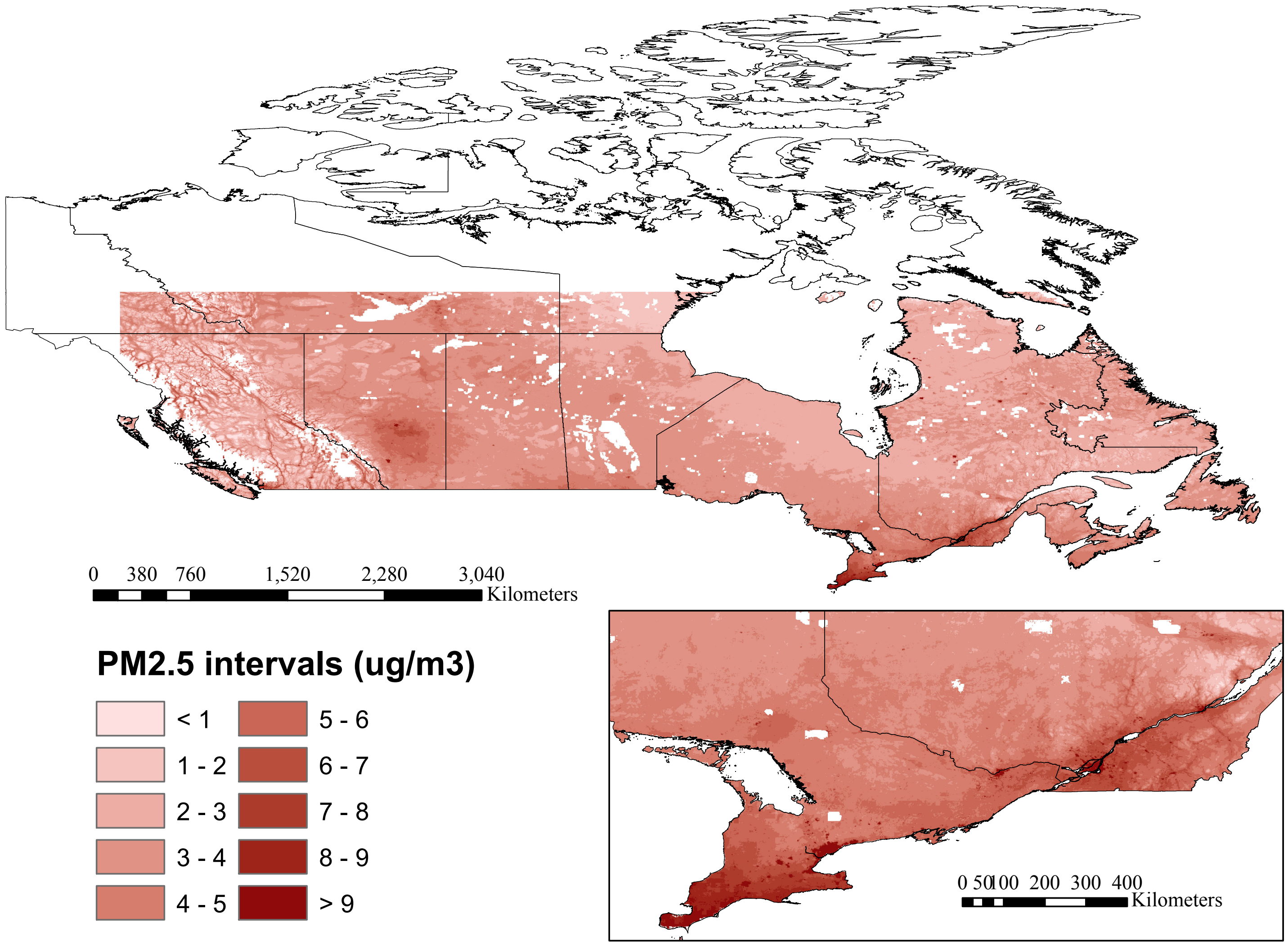

Supplemental Figure S4. Spatial variations in ambient O_x across Canada. Map created in ArcGIS Desktop 10.0. ESRI, Redlands, CA (http://desktop.arcgis.com/en/arcmap/).

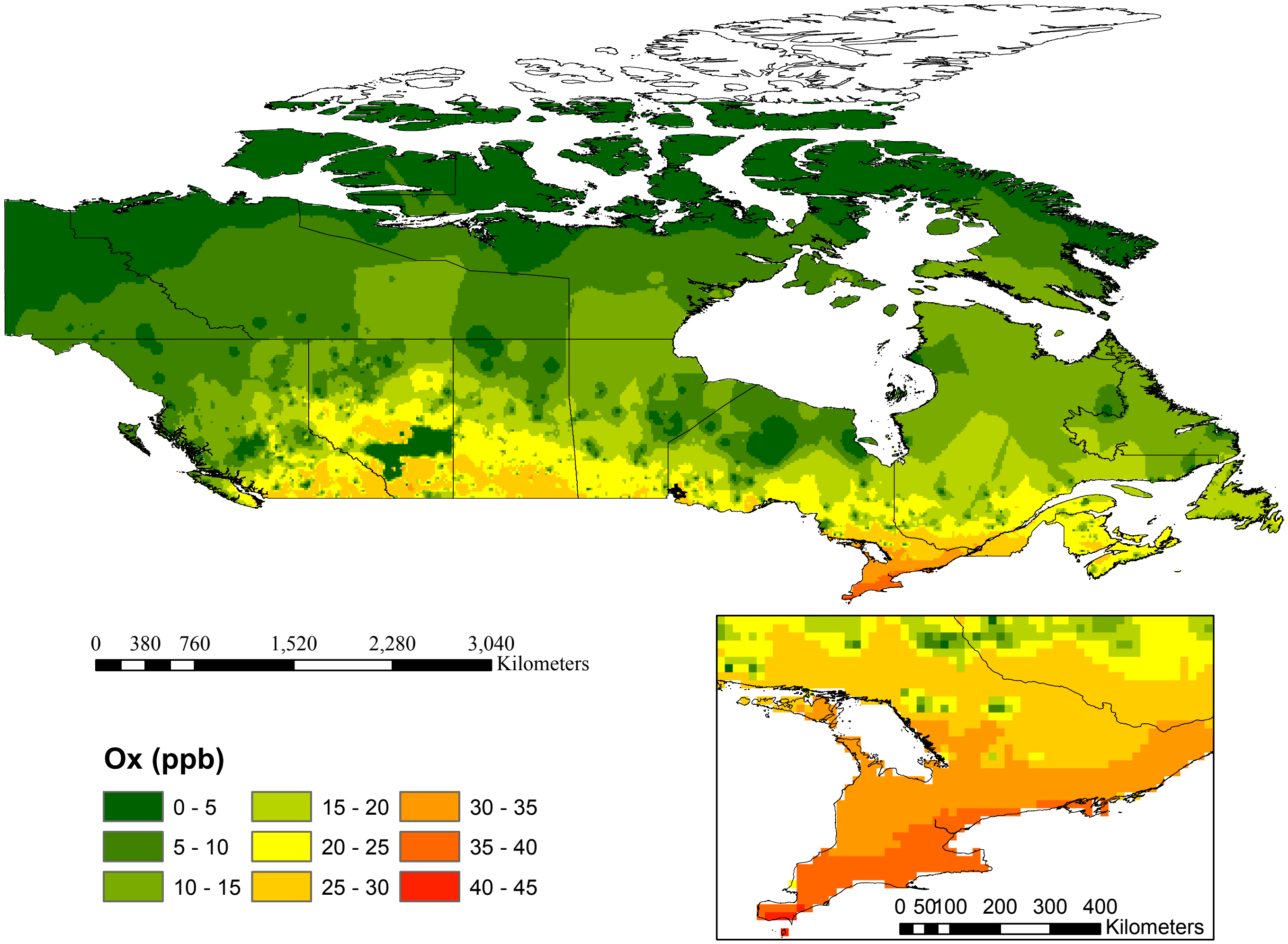

Supplement: Supplementary file 1 — Supplemental Material [file 41598_2017_16770_MOESM1_ESM.pdf]
